# Study on the double-edged sword effect of gen AI on college student entrepreneurial performance: evidence from the CICSIC

**DOI:** 10.3389/fpsyg.2026.1909353

**Published:** 2026-08-05

**Authors:** Shujun Yu, Yongkang Xu, Xu Han

**Affiliations:** 1 Institute of Innovation Management and Talent Development, Wuhan University of Science and Technology, Wuhan, China; 2 School of Management, Wuhan University of Science and Technology, Wuhan, China

**Keywords:** AI replacement perception, AI self-efficacy, CICSIC, cognitive appraisal theory of stress, college student entrepreneurial performance, Gen AI, value co-creation participation

## Abstract

Academic and science and technology competitions play a significant guiding role in fostering innovation and entrepreneurship capabilities among university students. Given the current focus on generative AI (Gen AI) applications and amid growing concerns about technological replacement, it is of great significance to explore the impact of Gen AI on students' entrepreneurial performance in competitions. Taking the China International College Student Innovation Competition (CICSIC) as a case study, this research draws upon cognitive appraisal theory of stress. It uses AI self-efficacy and AI replacement perception to establish a bridge for the mechanism through which Gen AI influences college student entrepreneurial performance. A three-wave time-lagged questionnaire survey was conducted among full-time undergraduate and postgraduate students participating in CICSIC entrepreneurial projects, and 376 valid observations were obtained. The hypotheses were tested using regression analysis, bootstrap mediation analysis, and moderation analysis. The results reveal that Gen AI has a significant positive impact on college student entrepreneurial performance, while AI self-efficacy and AI replacement perception play a double-edged role in this relationship. Value co-creation participation enhances the positive impact of AI self-efficacy on entrepreneurial performance and mitigates the negative impact of AI replacement perception. This study contributes to research on Gen AI in entrepreneurship education by revealing the challenge-oriented and threat-oriented appraisal mechanisms through which Gen AI influences students' entrepreneurial outcomes. The findings also suggest that universities and competition advisors should promote responsible human–AI collaboration while reducing students' perceived replacement threat.

## 1 Introduction

The CICSIC is an innovation and entrepreneurship contest jointly organized by the Ministry of Education, the United Front Work Department of the CPC Central Committee, and other departments. The competition originated from the China International College Students' “Internet+” Innovation and Entrepreneurship Competition (CICSIEC), launched in 2015, and was renamed its current title in 2023. Serving as a core practical platform for implementing the 2024–2035 Master Plan on Building China into a Leading Country in Education (Ministry of Education of the People's Republic of China, 2025), it undertakes an educational function characterized by “boosting teaching through competitions, stimulating innovation through competitions, and integrating industry and education.” It is also a youth innovation practice platform with the broadest disciplinary coverage and the highest level of international participation in China (Shi and Li, 2026). The 2024 competition attracted 5.14 million projects and 20.836 million participants from 5,406 universities in 153 countries and regions. A total of 470 projects were awarded gold medals, facilitating industry-education collaboration worth approximately 9.86 billion dollars. Included in the official directory of national college student competitions issued by the Ministry of Education of the People's Republic of China, the CICSIC has become one of the key indicators for evaluating the effectiveness of innovation and entrepreneurship education in higher education institutions. The competition consists of five major tracks: the Higher Education Main Track, the “Red Journey for Youth Dreams” Track, the Vocational Education Track, the Industry-Specific Track, and the Seedling Track. Covering all major disciplinary categories, including humanities, sciences, engineering, agriculture, medicine, and business, The competition requires participating teams to independently complete the full process of opportunity identification, interdisciplinary resource integration, business model design, market research, business plan writing, and project roadshow defense. By closely simulating the complete chain of real entrepreneurial practice, the CICSIC naturally provides an ideal research context for observing college students' use of AI tools, entrepreneurial behavior, and entrepreneurial performance.

Since ChatGPT brought Gen AI to broad public attention in 2022, Gen AI has been increasingly applied in higher education (Shi and Li, 2026). According to the competition notice issued by the Ministry of Education, the “AI+” projects under the Higher Education Main Track of the CICSIC 2025 further focus on the deep integration of AI with the development of all sectors of the economy and society, empowering the intelligent transformation and upgrading of industries across the board. The establishment of the “AI+” special track aims to actively respond to the development needs of new quality productive forces and to guide young students in the appropriate use of digital tools, including Gen AI, to empower innovation projects (Ministry of Education of the People's Republic of China, 2025). Meanwhile, the CICSIC has responded proactively to the rise of Gen AI with enthusiasm. Competition documents explicitly state that this track centers on the in-depth integration of artificial intelligence technologies with economic and social sectors, including the real economy, people's livelihood services, advanced manufacturing, modern agriculture, and digital cultural tourism. It guides participating teams to conduct project research and development based on Gen AI technologies such as large language models, computer vision, and intelligent algorithms, so as to advance intelligent transformation and industrial upgrading across all industries (China International College Students' Innovation Competition, 2025). To date, this has helped foster a new landscape of higher-quality entrepreneurship and employment for university graduates (Shi and Li, 2026).

Thus, within the context of the CICSIC, does Gen AI influence the entrepreneurial performance of university students? Previous research on the mechanisms influencing college student entrepreneurial performance has largely approached the topic from the perspective of the external environment, such as innovation and entrepreneurship education (Huang Y. et al., 2024), perceptions of entrepreneurial policy (Zhu and Yang, 2024), and the entrepreneurial ecosystem (Nie et al., 2025). Theoretical and empirical analyses have shown that the excessive integration of Gen AI can have both positive and negative effects: although it may enhance entrepreneurial performance (Huang Y. et al., 2024), it may also reduce students' creativity and critical thinking skills (Chen et al., 2024; Lei et al., 2025), increase technological dependence and anxiety (Huang S. et al., 2024), and contribute to homogenized content and academic misconduct (Shata and Hartley, 2025). However, three research gaps remain. First, prior studies have mainly examined Gen AI in general learning, academic writing, digital literacy, or technology acceptance contexts, while relatively little attention has been paid to its role in competition-based entrepreneurship education. Second, existing research often emphasizes the enabling effect of AI but insufficiently explains why Gen AI may simultaneously generate positive and negative psychological consequences for student participants through cognitive appraisal theory of stress. Third, although human–AI collaboration has been widely discussed (Xie et al., 2021), limited research has examined how value co-creation participation shapes the relationship between students' AI-related appraisals and entrepreneurial performance in team-based competition settings. Addressing these gaps, this study examines the double-edged mechanism through which Gen AI influences college students' entrepreneurial performance in the CICSIC context (Li et al., 2025b; Huang and Wang, 2025). Therefore, within the context of the CICSIC, there is a need for a systematic theoretical analysis of the impact of Gen AI on college student entrepreneurial performance and the mechanisms through which it operates.

In innovation and entrepreneurship competitions, the resources, opportunities, and team framework summarized by Timmons to explain the formation of entrepreneurial performance, and students' entrepreneurial activities typically involve opportunity identification, resource integration, and team collaboration (Timmons, 2010). This contextual understanding helps explain why Gen AI may be relevant to competition-related entrepreneurial tasks. However, the main theoretical focus of this study is not on the theory itself but on the psychological mechanisms through which students appraise and respond to Gen AI use (Lazarus and Folkman, 1987). Therefore, this study uses the cognitive appraisal theory of stress as the primary theoretical lens to explain the double-edged mechanisms of Gen AI, while task–technology fit theory and constructivist perspectives are used as supplementary explanations for the direct task-related effect and the moderating role of value co-creation participation (Goodhue and Thompson, 1995; Piaget and Inhelder, 1958).

The theoretical contributions of this study are as follows: Firstly, this study explores the impact of Gen AI on college student entrepreneurial performance within the context of the CICSIC, and in response to the call by Malik et al. for further research on the dual parallel mediation mechanisms underlying AI-related perceptions (Malik et al., 2022; Lazarus and Folkman, 1987), then establishes a relevant theoretical model. Secondly, drawing on the resources, opportunities, and team framework summarized by Timmons to explain the formation of entrepreneurial performance (Timmons, 2010), using cognitive appraisal theory of stress as the primary theory to explain the double-edged psychological mechanism through which Gen AI influences college student entrepreneurial performance. Specifically, AI self-efficacy is conceptualized as a challenge-oriented appraisal outcome, reflecting students' perceived capability and confidence in using Gen AI to cope with competition-related entrepreneurial tasks. AI replacement perception is conceptualized as a threat-oriented appraisal outcome, reflecting students' perceived risk that Gen AI may weaken their competence, task contribution, team role, or future entrepreneurial value (Lazarus and Folkman, 1987). Thirdly, this study extends research on Gen AI in higher education from general learning and academic support contexts to competition-based entrepreneurship education. By focusing on the CICSIC, this study explains how Gen AI may influence students' entrepreneurial growth and innovation performance in authentic entrepreneurial project tasks.

## 2 Literature review and hypotheses

### 2.1 Literature review

#### 2.1.1 The double-edged psychological mechanism: cognitive appraisal theory of stress

The growing use of Gen AI in innovation and entrepreneurship competitions has provided student teams with new forms of technological support. However, this technology may also introduce uncertainty, anxiety, and perceived threats related to students' competence, team roles, and future entrepreneurial value.

Lazarus and Folkman, 1987 first proposed the cognitive appraisal theory of stress, which posits that stress is not caused by the event itself, but rather arises from the cognitive appraisal process that individuals undergo when confronted with a stressor, progressing from primary appraisal to secondary appraisal and coping strategies (Lazarus and Folkman, 1987). In this framework, primary appraisal refers to an individual's assessment of the relevance of a stressor to their wellbeing based on internal conditions and the external environment, whereas secondary appraisal refers to the coping responses adopted after evaluating the internal and external resources available to address the stressor (Yang and Jiang, 2025). These concepts not only explain how individuals assess problems and determine appropriate responses under stress but also illustrate the theoretical transmission process (Li et al., 2025a).

Accordingly, as the core theoretical lens, the cognitive appraisal theory of stress is used to explain students' differentiated psychological responses to Gen AI as a potential technological stressor in the CICSIC context. This study focuses on two appraisal-related outcomes that are theoretically central to the double-edged effect of Gen AI (Yang and Jiang, 2025). AI self-efficacy is interpreted as a challenge-oriented appraisal outcome because it reflects students' perceived capability and confidence in using Gen AI to cope with competition-related entrepreneurial tasks. By contrast, AI replacement perception is interpreted as a threat-oriented appraisal outcome because it reflects students' perceived risk that Gen AI may substitute their task value, weaken their team role, or undermine their future entrepreneurial development. Therefore, cognitive appraisal theory provides the core explanatory logic for understanding why Gen AI may simultaneously generate enabling and constraining effects on college student entrepreneurial performance.

### 2.2 Hypotheses

#### 2.2.1 The impact of Gen AI on college student entrepreneurial performance

Gen AI refers to a class of algorithm- and model-based technologies that generate high-quality content creatively by learning from massive amounts of data to meet diverse user needs (Yang and Jiang, 2025). Given the survey-based design of this study, Gen AI is operationalized from the perspective of students' perceived task-related support (Yang and Jiang, 2025). Its key characteristics include complementarity, utilization, heterogeneity, and value-creation benefit (Deitz et al., 2010; Lu et al., 2016; Ainuddin et al., 2007; Liang et al., 2021). Therefore, the present study examines Gen AI as perceived by student participants in terms of its task-related support and resource value. It captures individual students' perceived resource value of Gen AI in completing CICSIC-related entrepreneurial tasks, such as opportunity identification, business-plan writing, market analysis, prototype design, financial analysis, and project presentation.

In the field of entrepreneurship research, entrepreneurial performance, first proposed by Sandberg, is regarded as a key indicator for measuring entrepreneurial outcomes and effectiveness (Sandberg and Hofer, 1987). Haber and Reichel (2005) were among the first to divide entrepreneurial performance into subjective and objective criteria. The former includes development, growth, and business strength, while the latter includes market share, firm size, and profitability relative to competitors (Haber and Reichel, 2005). However, traditional measures of entrepreneurial performance are often based on objective indicators such as market share, operating profit, and firm survival scale, which are more applicable to mature market-oriented entrepreneurial entities (Zhu et al., 2022). By contrast, college students' entrepreneurship is typically characterized by early-stage development and competition-driven entrepreneurial activities. Most students' entrepreneurial projects have not yet generated stable revenue, market share, or other quantifiable objective business data. Therefore, objective performance indicators have significant limitations in this context. Subjective criteria, such as innovation quality, practical effectiveness, growth potential, and competitive performance, are more suitable for questionnaire-based research (Liu et al., 2019). Accordingly, scholars generally argue that college students' entrepreneurial performance refers to the comprehensive entrepreneurial outcomes and innovative achievements generated through the transformation of students' entrepreneurial capabilities into entrepreneurial practice. In other words, it reflects the outcome benefits produced during the process by which college students convert entrepreneurial competence into practical entrepreneurial activities, mainly including entrepreneurial growth performance and entrepreneurial innovation performance (Yang and Yang, 2017; Xu et al., 2017). In addition, objective competition indicators, such as award level, advancement status, judges' scores, or project ranking, were not used in this study. Due to different tracks and competition levels, the CICSIC may apply different evaluation criteria, making direct comparison difficult. And many participating projects are still at an early stage and have not generated stable markets or financial outcomes. Therefore, self-reported entrepreneurial performance is more suitable for capturing individual students' perceived entrepreneurial growth and innovation outcomes in this context (Xu et al., 2017; Mu et al., 2020).

According to the task-technology fit theory (Goodhue and Thompson, 1995), technology facilitates task performance when it fits users' actual task requirements (Düz and Kavak, 2026). In the context of the CICSIC, collaboration between Gen AI and student teams can support contestants in efficiently producing business plans, PowerPoint presentations, design prototypes, and market and financial analyses. In doing so, Gen AI can reduce time and resource costs while allowing students to devote greater cognitive resources to creative discussion, critical thinking, and the practical application of innovation and entrepreneurship knowledge. In addition, its capacity to integrate interdisciplinary knowledge provides students with a broader innovation perspective and enables them to identify business opportunities across multiple domains. As a result, Gen AI can more effectively enhance the innovation quality, practical effectiveness, growth potential, and competitive performance of college students' entrepreneurial activities.

Therefore, the following hypothesis is proposed:

H1: Gen AI has a significant positive impact on college student entrepreneurial performance.

#### 2.2.2 The mediating role of AI self-efficacy

AI self-efficacy refers to students' belief in their capability to use Gen AI effectively to complete learning, innovation, and entrepreneurship-related tasks. It reflects students' confidence in operating Gen AI tools, obtaining useful information, evaluating AI-generated content, and applying AI-assisted outputs to competition-related entrepreneurial tasks (Erol et al., 2025; Chun et al., 2025). From the perspective of cognitive appraisal theory of stress, AI self-efficacy can be understood as a challenge-oriented appraisal outcome. When students encounter Gen AI as a potential stressor, they first engage in primary appraisal by evaluating whether it is relevant to their wellbeing and whether it is likely to facilitate or hinder project progress and personal capability enhancement during the competition (Lazarus and Folkman, 1987). When students perceive Gen AI as helpful for completing tasks more efficiently and regard it as a collaborative “quasi-colleague” (Chun et al., 2025), they are more likely to form positive attributions and believe that they can accomplish specific tasks through interaction with Gen AI, thereby exhibiting higher AI self-efficacy.

Students with higher AI self-efficacy are more likely to believe that they can address challenging innovation and entrepreneurship tasks with the support of Gen AI in CICSIC. As a result, they tend to devote greater time and effort to exploring Gen AI's functions, integrating relevant resources, and improving usage efficiency. This process can lead to more comprehensive and innovative business plans and provide a stronger foundation for subsequent entrepreneurial project implementation. In addition, interaction with Gen AI allows students to refine business model ideas, correct grammatical problems in business plans, improve document formatting, and complete repetitive basic market analyses. Through these experiences, students accumulate competence and confidence, creating a virtuous cycle that further reinforces their AI self-efficacy.

Therefore, the following hypothesis is proposed:

H2: AI self-efficacy mediates the relationship between Gen AI and college student entrepreneurial performance in a positive manner.

H2a: Gen AI has a significant positive influence on AI self-efficacy.

H2b: AI self-efficacy has a significant positive effect on college student entrepreneurial performance.

#### 2.2.3 The mediating role of AI replacement perception

AI replacement perception refers to an individual's perception that AI may replace their profession or negatively affect their career prospects (Zhang et al., 2024). In the CICSIC context, if students perceive Gen AI as a highly disruptive technology and believe that the content it produces threatens their position and value within the team, they may develop negative emotions such as anxiety, frustration, and panic, thereby forming AI replacement perceptions. From the perspective of cognitive appraisal theory of stress, AI replacement perception can be interpreted as a threat-oriented appraisal outcome. It reflects students' perception that Gen AI may threaten their competence, team status, and future entrepreneurial value. When students perceive Gen AI as having substitutive potential or comparative advantages in core domains such as technical design, coding, or creative work, they may regard it as a stressor that threatens their team status and academic value (Lazarus and Folkman, 1987). Once such an appraisal is formed, students may associate the perceived threat of replacement with diminished self-worth and organizational self-esteem. As a result, they may experience frustration rooted in technological dependence and exhibit avoidance behaviors, including resistance to or rejection of Gen AI (Paluch et al., 2022; Bai et al., 2025). Moreover, AI replacement perception may blur students' judgment of their own professional value and core competitiveness, weaken their identification with traditional skills and assigned team roles, and foster passive coping attitudes such as “Buddhist-style” or “lying-flat” responses, all of which are detrimental to entrepreneurial performance (Yan et al., 2024; Liu and Qin, 2025). It may also contribute to technical misconduct, such as the use of fabricated citations generated by AI, and reduce students' willingness to engage with Gen AI (Park and Park, 2025). In turn, this may constrain the effective integration of technological resources and cause teams to miss important innovation opportunities, thereby undermining long-term entrepreneurial competitiveness.

Consequently, the impact of Gen AI technology on university student participants is not simply a case of ‘the more, the better.' AI replacement perception may reduce entrepreneurial performance through several mechanisms. First, when students believe that Gen AI may replace their task contributions, they may reduce their task engagement and become less willing to invest time and effort in project development (Klingbeil et al., 2024). Second, perceived replacement threat may weaken students' sense of role value within the team, making them feel that their knowledge, creativity, or professional skills are less important (Chen et al., 2019; Yang and Jiang, 2025). Third, such perceptions may reduce team participation and communication, because students may avoid tasks in which Gen AI appears to perform better (Zong et al., 2025). Finally, AI replacement perception may constrain creative thinking by inducing anxiety, self-doubt, and passive coping (Düz and Kavak, 2026). These psychological and behavioral responses may ultimately undermine students' entrepreneurial growth and innovation performance.

Therefore, the following hypothesis is proposed:

H3: AI replacement perception mediates the relationship between Gen AI and college student entrepreneurial performance.

H3a: Gen AI significantly and positively influences AI replacement perception.

H3b: AI replacement perception has a significant negative effect on college student entrepreneurial performance.

#### 2.2.4 The moderating role of value co-creation participation

Value co-creation participation derives from value co-creation theory, which originally conceptualized value as jointly created by multiple actors rather than produced unilaterally by firms (Prahalad and Ramaswamy, 2004). With the deepening of human–AI collaboration, AI has increasingly been incorporated into the value creation process as a “quasi-colleague” (Li et al., 2023; Chun et al., 2025). In the context of the CICSIC, value co-creation participation can be understood along two dimensions: human–machine value co-creation and team value co-creation (Ma et al., 2024). The two dimensions are theoretically connected because both reflect students' active involvement in value creation through interaction, feedback, resource integration, and task collaboration. Human–machine value co-creation provides technological support and idea generation, whereas team value co-creation provides interpersonal support, role clarification, and resource coordination. Together, they form a collaborative context that may strengthen the positive effect of AI self-efficacy and buffer the negative effect of AI replacement perception.

Students' engagement with Gen AI is not an isolated individual practice; rather, it is embedded in a broader process of value co-creation involving technological tools and peer collaboration. This interaction-oriented mechanism makes constructivist theory relevant for understanding the role of value co-creation participation.

Constructivist thought originates in the work of Piaget (Piaget and Inhelder, 1958). Building on this foundation, Vygotsky (1978) further developed constructivist learning theory. In particular, he emphasized the central role of interaction and collaboration in learning, suggesting that these processes are crucial for knowledge construction, knowledge transfer, and social development (Vygotsky, 1978). Accordingly, the constructivist perspective holds that knowledge is not passively received through rote instruction but is actively constructed through social interaction and linguistic communication (Kegan, 1982; O'Donnell et al., 2012; Saleem et al., 2021). Based on this perspective, Constructivist Theory reveals that university students can lower barriers to resource access through human–machine value co-creation, while benefiting from the support of team value co-creation from team members and supervising lecturers. Thus, facilitating the effective acquisition, comprehension, evaluation, and application of interdisciplinary innovation- and entrepreneurship-related knowledge strengthens the linkage between new and prior knowledge and supports the development of innovation and entrepreneurship projects across diverse contexts. From a constructivist perspective, both forms of co-creation facilitate knowledge construction, knowledge sharing, and role development through interaction and communication (Saleem et al., 2021). High levels of human–machine value co-creation enable students to use Gen AI to bridge knowledge gaps, lower learning barriers, and improve the efficiency and quality of task completion. At the same time, high levels of team value co-creation strengthen resource sharing, role clarity, emotional support, and trust among members (Zhao et al., 2022). Together, these processes can enhance students' confidence in using Gen AI, reduce the negative consequences of technological dependence and role ambiguity, and create a supportive environment for the appropriate use of Gen AI in innovation and entrepreneurship activities.

H4a: Value co-creation participation positively moderates the relationship between AI self-efficacy and college student entrepreneurial performance. The higher the level of value co-creation participation, the stronger the positive relationship between AI self-efficacy and college student entrepreneurial performance.

H4b: Value co-creation participation positively moderates the relationship between AI replacement perception and college student entrepreneurial performance. The higher the level of value co-creation participation, the weaker the negative relationship between AI replacement perception and college student entrepreneurial performance.

#### 2.2.5 Research model

**Figure 1:**
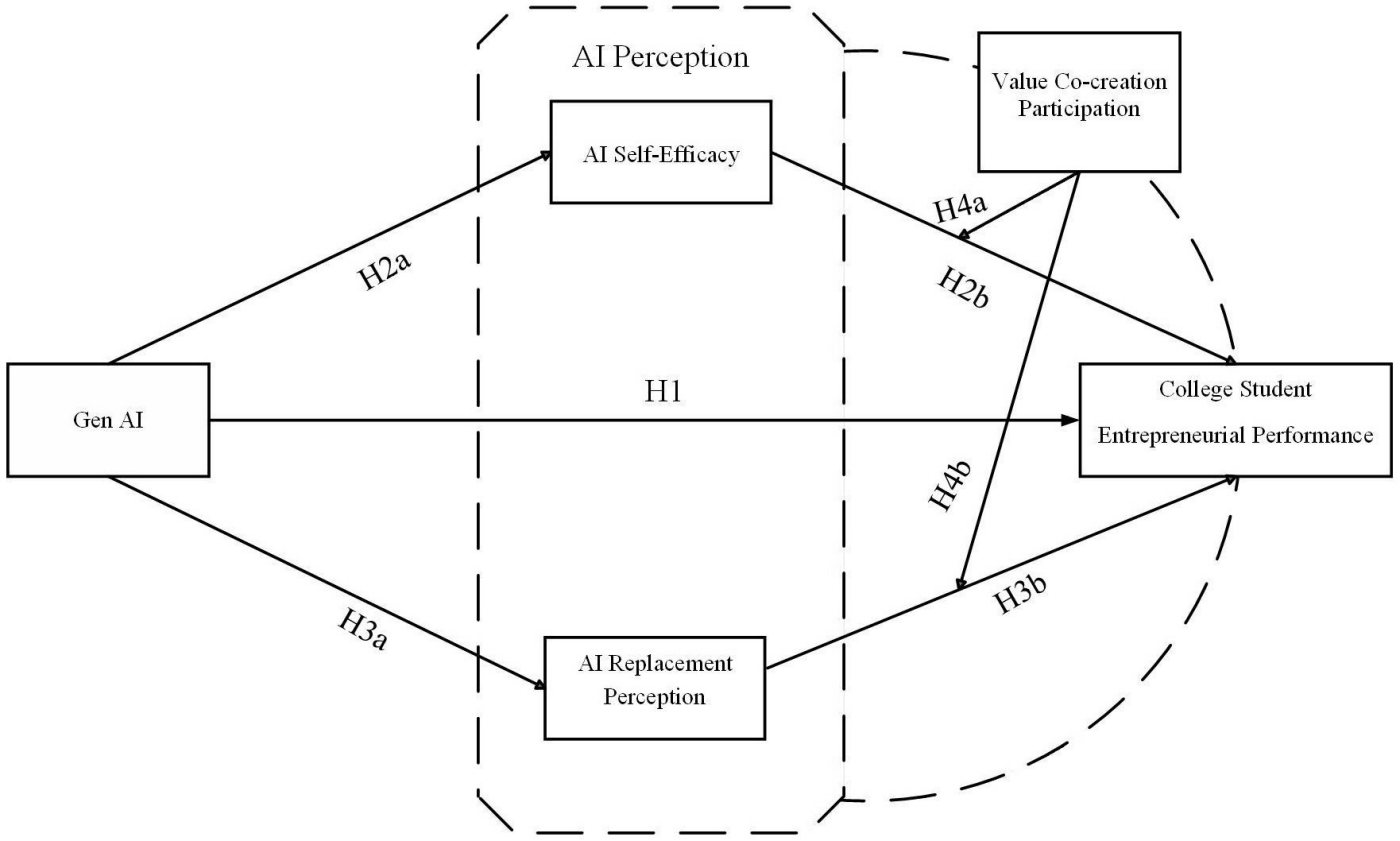
Research model.

In summary, a research model linking Gen AI and college student entrepreneurial performance is constructed, as shown in Figure 1.

## 3 Research design

### 3.1 Study design and data collection

This study targeted full-time undergraduate and postgraduate students from higher education institutions participating in the entrepreneurship category of the CICSIC, excluding part-time students and teaching staff. All focal variables, including Gen AI, AI self-efficacy, AI replacement perception, value co-creation participation, and college student entrepreneurial performance, were measured as individual students' self-reported perceptions. The sample covered projects from the Higher Education Main Track, the Industry-Specific Track, the “Youth Red Dream Journey” Track, and the Vocational Education Track, while excluding the Seedling Track. To mitigate common method bias, a three-wave time-lagged design was adopted in combination with snowball sampling. Participants received small monetary incentives after completing each survey wave: 1 yuan for Wave 1, 3 yuan for Wave 2, and 5 yuan for Wave 3. The total incentive cost was 3,482 yuan. These incentives were paid from the authors' personal funds and did not constitute external research funding. Each wave lasted 15 days, and the respondents were mainly drawn from participating teams at universities in China.

To enhance measurement validity, the study first reviewed prior literature on the relationship between Gen AI and entrepreneurial performance and then conducted interviews and pilot surveys with team members who had participated in competitions at the university, provincial, and national levels. The pilot results suggested that team members frequently used Gen AI to support task completion, but held mixed views about it. Established scales from prior domestic and international studies were then adapted with input from relevant faculty members and students. The final questionnaire was administered online, which helped reduce incomplete responses (Liang and Xie, 2018).

Before completing the questionnaire, respondents were asked to confirm that they were full-time undergraduate or postgraduate students and had participated in at least one CICSIC-related innovation and entrepreneurship project. The questionnaire also collected information on competition track, competition level, and task responsibility within the team to further verify respondents' participation experience. Respondents who did not meet the inclusion criteria or failed to complete all three waves were excluded from the final sample.

In the formal survey, Wave 1 collected data on Gen AI and the control variables, including gender, year of study, competition track, and task responsibilities within the team, yielding 417 valid responses. Two weeks later, Wave 2 collected data on AI self-efficacy, AI replacement perception, and value co-creation participation, yielding 395 valid responses. Two weeks after that, Wave 3 collected data on college student entrepreneurial performance, yielding 376 valid questionnaires and an overall effective response rate of 82.41%. Sample analysis showed that 52.93% of respondents were male, 63.29% were in their second to fourth years of study, and most teams participated in the Higher Education Main Track (25.60%) or the Industry Track (31.64%). In addition, 87.20% of teams were at the university preliminary or provincial semi-final level, and the most common team roles were business operations, market analysis, finance, and legal affairs (33.82%).

### 3.2 Variable measurement

To ensure the reliability of the questionnaire, all scales used in this study are well-established scales from both domestic and international sources, suitable for the Chinese context. All items were measured using a 5-point Likert scale.

Gen AI: The Gen AI scale was adapted from prior studies and included four dimensions with 11 items. Specifically, the complementarity dimension was adapted from Deitz et al. (2010), for example, “The effective resources provided by generative artificial intelligence supplement my learning and competition-related entrepreneurial tasks.” The utilization dimension was adapted from Lu et al. (2016), for example, “By using Gen AI, I can easily obtain the resources I need.” The heterogeneity dimension was adapted from Ainuddin et al. (2007), for example, “Compared with other technologies, the resources provided by Gen AI are difficult to replace.” The value-creation benefit dimension was adapted from Liang et al. (2021), for example, “Gen AI saves me time and effort when completing learning and competition-related tasks.” Although the original scales were developed mainly in commercial contexts, these dimensions reflect general resource-evaluation attributes, and they are applicable to the present individual-level setting after contextual adaptation.

College Student Entrepreneurial Performance: The scale was adapted from prior studies and included two dimensions with six items. Specifically, the entrepreneurial growth performance dimension was adapted from Yang and Yang (2017), for example, “In the process of innovation and entrepreneurship, I used the content I learned from entrepreneurship education, strengthened my innovation awareness and entrepreneurial ability, and improved my overall quality.” The entrepreneurial innovation performance dimension was adapted from Xu et al. (2017), for example, “The creative and technological achievements I have achieved in the process of innovation and entrepreneurship have a high success rate of being transformed into products and are expected to be utilized by schools or the market.”

Value co-creation participation: The scale was adapted from Ma et al. (2024) and included two dimensions with eight items. Specifically, the team complementary value co-creation dimension captures students' participation in team-based collaboration and resource support, for example, “I can use Gen AI to jointly develop intelligent entrepreneurial projects with my team.” The human-machine collaborative value co-creation dimension captures students' participation in value creation through interaction with Gen AI and intelligent services, for example, “Gen AI can present us with creative ideas.”

AI Self-Efficacy: The AI self-efficacy scale was adapted from Zheng et al. (2024) and included one dimension with four items. This scale measures students' confidence in using Gen AI to complete learning and competition-related tasks. For example, “I am confident in using Gen AI even if no one is assisting.”

AI Replacement Perception: The AI replacement perception scale was adapted from Brougham and Haar (2018) and included one dimension with four items. For example, “I think that the work or tasks I take on in study and competition may be replaced by AI technology.”

## 4 Results

### 4.1 Reliability, validity, and common method bias tests

#### 4.1.1 Reliability tests

In this study, Cronbach's alpha coefficients were calculated using Stata 15.0. The Cronbach's alpha coefficients for the Gen AI scale, the college student entrepreneurial performance scale, the value co-creation participation scale, the AI self-efficacy scale, and the AI replacement perception scale were 0.938, 0.891, 0.920, 0.882, and 0.816, respectively. As all coefficients exceeded 0.8, the results indicate that the scales demonstrated good reliability.

#### 4.1.2 Validity testing

First, the KMO measure and Bartlett's test of sphericity were conducted using Stata 15.0. The results showed that the KMO value was 0.903, the approximate chi-square value was 8,481.653, the degrees of freedom were 528, and the significance level was 0.000, indicating that the data were suitable for confirmatory factor analysis. In addition, the dimensions of each variable were clearly distinguishable. Second, although the data were collected in three separate waves, all variables were self-reported by the same respondents and may therefore be affected by common method bias. To examine this issue, confirmatory factor analysis was conducted in Amos 24.0 using a multi-factor model. As shown in Table 1, the five-factor model exhibited a good fit to the data (χ^2^/df = 2.443, RMSEA = 0.062, TLI = 0.907, CFI = 0.915, SRMR = 0.065), and it outperformed the alternative models, suggesting that the five study variables had good discriminant validity. Although the TLI and CFI values of the five-factor model were close to the conventional threshold of 0.90, they still met the acceptable criteria for model fit. This relatively borderline fit may be related to the complexity of the measurement model, which included five latent constructs and multiple dimensions, as well as the fact that all variables were measured using self-reported questionnaire data in a competition-based educational context. More importantly, the five-factor model showed substantially better fit than the alternative four-factor, three-factor, two-factor, and single-factor models, supporting the discriminant validity of the focal constructs.

**Table 1:** Confirmatory factor analysis.

Measurement model and indicators	Factor structure	χ^2^	df	χ^2^/df	RMSEA	TLI	CFI	SRMR
Five-factor model	GAI, CSEP, VCP, AISE, AIRP	1,184.761	485	2.443	0.062	0.907	0.915	0.065
Four-factor model	GAI, CSEP, VCP, AISE + AIRP	1,820.756	489	3.723	0.085	0.825	0.838	0.182
Three-factor model	GAI, CSEP, VCP + AISE + AIRP	2,698.021	492	5.484	0.109	0.712	0.732	0.097
Two-factor model	GAI, CSEP + VCP + AISE + AIRP	3,601.496	494	7.290	0.130	0.596	0.622	0.1212
Single-factor model	GAI+CSEP+VCP+ AISE+AIRP	5,223.301	495	10.552	0.160	0.387	0.425	0.1246

GAI denotes Gen AI, CSEP denotes College Student Entrepreneurial Performance, VCP denotes Value Co-creation Participation, AISE denotes AI Self-Efficacy, and AIRP denotes AI Replacement Perception.

#### 4.1.3 Common method bias test

Harman's single-factor test was first used to examine common method bias. The results showed that the first factor explained 30.85% of the total variance, which is below the 40% threshold. To further assess common method bias, this study conducted an unmeasured latent method factor test. The results of the unmeasured latent method factor test are summarized in Table 2. Specifically, a common latent method factor was added to the baseline five-factor confirmatory factor analysis model, and all measure.ment items were allowed to load on both their theoretical constructs and the common method factor. The baseline five-factor model showed acceptable fit: χ^2^/df = 2.443, RMSEA = 0.062, TLI = 0.907, CFI = 0.915, and SRMR = 0.065. After adding the common latent method factor, the model fit was χ^2^/df = 2.432, RMSEA = 0.062, TLI = 0.908, CFI = 0.916, and SRMR = 0.065. The changes in fit indices were small: Δχ^2^/df = −0.011, ΔRMSEA = 0.000, ΔTLI = 0.001, ΔCFI = 0.001, and ΔSRMR = 0.000. These results indicate that the inclusion of the common method factor did not substantially improve model fit, suggesting that common method bias was not a serious concern.

**Table 2:** Unmeasured latent method factor.

Model	χ^2^/df	RMSEA	TLI	CFI	SRMR
Five-factor model	2.443	0.062	0.907	0.915	0.065
Five-factor model + CMF	2.432	0.062	0.908	0.916	0.065
Difference	−0.011	0.000	0.001	0.001	0.000

CMF, common method factor. The common method factor was added to the baseline five-factor CFA model, with all measurement items loading on both their theoretical constructs and the common method factor. The common method factor was constrained to be orthogonal to the theoretical constructs. Difference values were calculated as the fit indices of the method-factor model minus those of the baseline five-factor model.

### 4.2 Descriptive statistics and correlation analysis

Descriptive statistics and correlation analyses were conducted using Stata 15.0. As shown in Table 3, Gen AI was found to be significantly positively correlated with college student entrepreneurial performance (*r* = 0.468, *p* < 0.01), AI self-efficacy (*r* = 0.392, *p* < 0.01), and AI replacement perception (*r* = 0.227, *p* < 0.01), AI self-efficacy (*r* = 0.543, *p* < 0.01) also showed a significant positive correlation with college student entrepreneurial performance, whereas AI replacement perception (*r* = −0.343*, p* < 0.01) exhibited a significant negative correlation with college student entrepreneurial performance. These empirical findings provide preliminary support for research hypotheses H1, H2, H2a, H2b, H3, H3a, and H3b, indicating that there are significant theoretical associations between the variables and providing a statistical basis for further analysis.

**Table 3:** Descriptive statistics and correlation analysis.

Variable measurement	*M*	*SD*	1	2	3	4	5	6	7	8	9	10
1. Gender	1.52	0.50	1									
2. Age	2.76	1.49	−0.018	1								
3. Track	2.54	1.10	−0.004	−0.057	1							
4. Level	1.54	0.70	0.021	−0.068	−0.035	1						
5. Tasks	2.58	0.92	0.009	0.021	0.022	0.024	1					
6. GAI	3.40	1.18	0.012	−0.015	0.048	0.024	0.104^*^	1				
7. AISE	3.54	1.18	−0.082	−0.058	0.034	−0.024	−0.060	0.392^**^	1			
8. AIRP	2.73	1.04	−0.010	0.014	0.109^*^	0.002	0.037	0.227^**^	−0.113^**^	1		
9. VCP	3.57	1.05	−0.016	−0.084	0.018	−0.022	0.106^*^	0.180^**^	0.388^**^	−0.269^**^	1	
10. CSEP	3.75	1.09	−0.08	−0.093	0.027	0.017	0.301^**^	0.468^**^	0.543^**^	−0.343^**^	0.341^**^	1

^**^indicates a 1% significance level (significant correlation), ^*^ indicates a 5% significance level (significant correlation).

### 4.3 Hypothesis testing

#### 4.3.1 Testing for main effects and mediating effects

Main effects were tested using Stata 15.0, and the results are reported in Table 4. Model (2) examined the effect of Gen AI on AI self-efficacy. The results showed that Gen AI had a significant positive effect on AI self-efficacy (β = 0.386, *P* < 0.001), supporting hypothesis H2a. Model (4) examined the effect of Gen AI on AI replacement perception. The results indicated that Gen AI had a significant positive effect on AI replacement perception (β = 0.201, *p* < 0.001), supporting hypothesis H3a. Model (6) examined the effect of Gen AI on college student entrepreneurial performance. The findings showed that Gen AI had a significant positive effect on college student entrepreneurial performance (β = 0.425, *P* < 0.001), thereby providing initial support for hypothesis H1. Model (7) further showed that AI self-efficacy had a significant positive effect on college student entrepreneurial performance (β = 0.388, *p* < 0.001), supporting hypothesis H2b. Model (8) showed that AI replacement perception had a significant negative effect on college student entrepreneurial performance (β = −0.497, *p* < 0.001), supporting hypothesis H3b. Taken together, these findings provide preliminary support for the mediating effects proposed in hypotheses H2 and H3.

**Table 4:** Main effect test.

Variables	(1)	(2)	(3)	(4)	(5)	(6)	(7)	(8)
	AISE	AISE	AIRP	AIRP	CSEP	CSEP	CSEP	CSEP
Gender	−0.196	−0.207^*^	−0.018	−0.023	−0.022	−0.034	0.046	−0.046
Age	−0.049	−0.045	−0.016	0.018	−0.068^*^	−0.064^*^	−0.047	−0.055^*^
Track	0.029	0.009	0.105^**^	0.096^**^	0.019	−0.002	−0.006	0.046
Level	−0.048	−0.063	0.014	0.006	0.013	−0.002	0.021	0.001
Task	0.135^*^	0.099	−0.071	−0.090	0.126^**^	0.085	0.047	0.041
GAI		0.386^***^		0.201^***^		0.425^***^	0.275^***^	0.525^***^
AISE							0.388^***^	
AIRP								−0.497^***^
R^2^	0.023	0.278	0.016	0.068	0.021	0.232	0.379	0.442
Δ R^2^	0.009	0.171	0.003	0.053	0.008	0.220	0.367	0.431
F	1.75	12.71^***^	1.23	4.49^***^	1.57	18.57^***^	32.03^***^	41.65^***^

^*^p < 0.10, ^**^p < 0.05, ^***^p < 0.01.

#### 4.3.2 Testing for mediating effects

The PROCESS procedure was employed with Bootstrap resampling (5,000 repetitions) to test the parallel mediating effects of AI self-efficacy and AI replacement perception on the relationship between Gen AI and college student entrepreneurial performance. The results are shown in Table 5.

**Table 5:** Mediation effect test.

Path	Effect Size	S.E.	LLCI	ULCI	Significance	Effect size
Indirect effect 1 (GAI → AISE → CSEP)	0.133	0.030	0.077	0.195	^***^	0.267
Indirect effect 2 (GAI → AIRP → CSEP)	−0.042	0.016	−0.076	−0.015	^***^	0.084
Total indirect effect	0.091	0.034	0.001	0.180	^***^	0.182
Direct effect	0.408	0.036	0.338	0.479	^***^	0.818
Total effect	0.499	0.050	0.339	0.659	^***^	

^***^p < 0.01.

As shown in Table 5, the total effect of Gen AI on college student entrepreneurial performance was significant (c = 0.499, *p* < 0.001), and the direct effect was also significant (c' = 0.408, p < 0.001), accounting for 81.8% of the total effect. Of the two indirect effects, “GAI → AISE → CSEP,” that is, indirect effect 1 (c1 = 0.133, *p* < 0.001), was significant, with a 95% confidence interval of [0.077, 0.195], which does not include zero, accounting for 26.7% of the total effect. “GAI → AIRP → CSEP,” that is, indirect effect 2 (c2 = −0.042, p < 0.001), was also significant, with a 95% confidence interval of [−0.076, −0.015], which does not include zero, accounting for 8.4% of the total effect. The total indirect effect was 0.091 and significant (p < 0.001), with a 95% confidence interval of [0.001, 0.18], which does not include zero, accounting for 18.2% of the total effect. Because the direct effect remained significant after including AI replacement perception, the mediation effect can be interpreted as partial mediation. Based on these results, hypotheses H1, H2, H3, H2a, H2b, H3a, and H3b were supported.

These results indicate that AI self-efficacy and AI replacement perception have significant mediating effects on the relationship between Gen AI and college student entrepreneurial performance.

#### 4.3.3 Moderation effect analysis

This study employed Stata 15.0 to examine the moderating effect of value co-creation participation. First, value co-creation participation, AI self-efficacy, and AI replacement perception were mean-centered, and interaction terms were created between AI self-efficacy and value co-creation participation, as well as between AI replacement perception and value co-creation participation. College student entrepreneurial performance was then entered as the dependent variable. As shown in Table 6, the explanatory power of the models improved progressively from M1 to M3, as indicated by the continuous increase in *R*^2^. In addition, the F-values for all models were significant at the 0.001 level, suggesting that the models were statistically significant overall. The interaction between AI self-efficacy and value co-creation participation had a significant positive effect on college student entrepreneurial performance (β = 0.101, *p* < 0.001), indicating that value co-creation participation positively moderates this relationship. Specifically, a higher level of value co-creation participation strengthens the positive effect of AI self-efficacy on college student entrepreneurial performance, supporting H4a. Similarly, the interaction between AI replacement perception and value co-creation participation also had a significant positive effect on college student entrepreneurial performance (β = 0.225, p < 0.001). This suggests that value co-creation participation weakens the negative effect of AI replacement perception on college students' entrepreneurial performance, thereby supporting H4b. Taken together, these findings support H4.

**Table 6:** Moderation effect test.

Variables	(1)	(2)	(3)
	CSEP	CSEP	CSEP
Gender	0.036	−0.034	0.022
Age	−0.034	−0.050^*^	−0.037
Track	−0.008	0.035	0.025
Level	0.034	0.043	0.071
Task	0.041	0.042	0.014
Gen AI	0.271^***^	0.519^***^	0.408^***^
AISE	0.342^***^		0.302^***^
AIRP		−0.386^***^	−0.318^***^
VCP	0.164^***^	0.164^***^	0.099^**^
AISE x VCP	0.101^**^		0.096^***^
AIRP x VCP		0.225^***^	0.256^***^
R^2^	0.407	0.499	0.584
Δ R^2^	0.393	0.487	0.571
F	27.95^***^	40.54^***^	46.48^***^

*AISE* × *VCP and AIRP* × *VCP denote the interaction terms constructed from the mean-centered variables*. ^*^p < 0.10, ^**^p < 0.05, ^***^p < 0.01.

**Figure 2:**
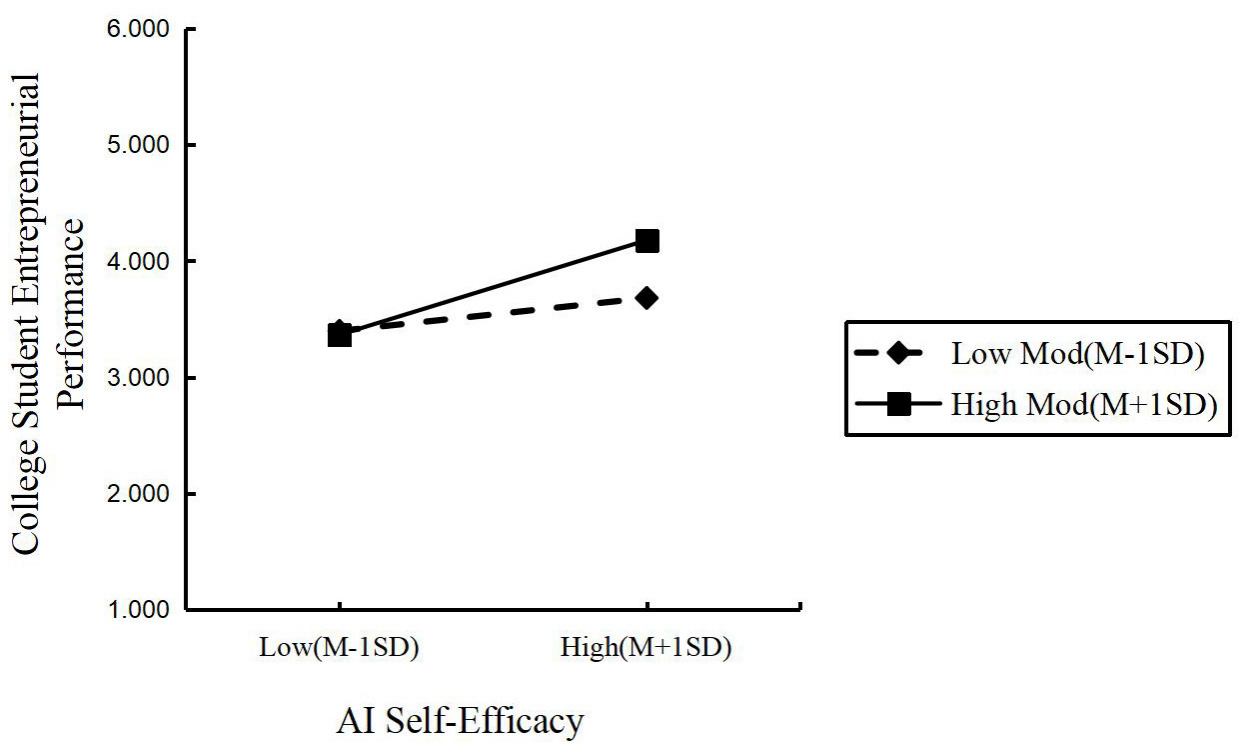
Moderating effect of VCP on the AISE–CSEP relationship.

**Figure 3:**
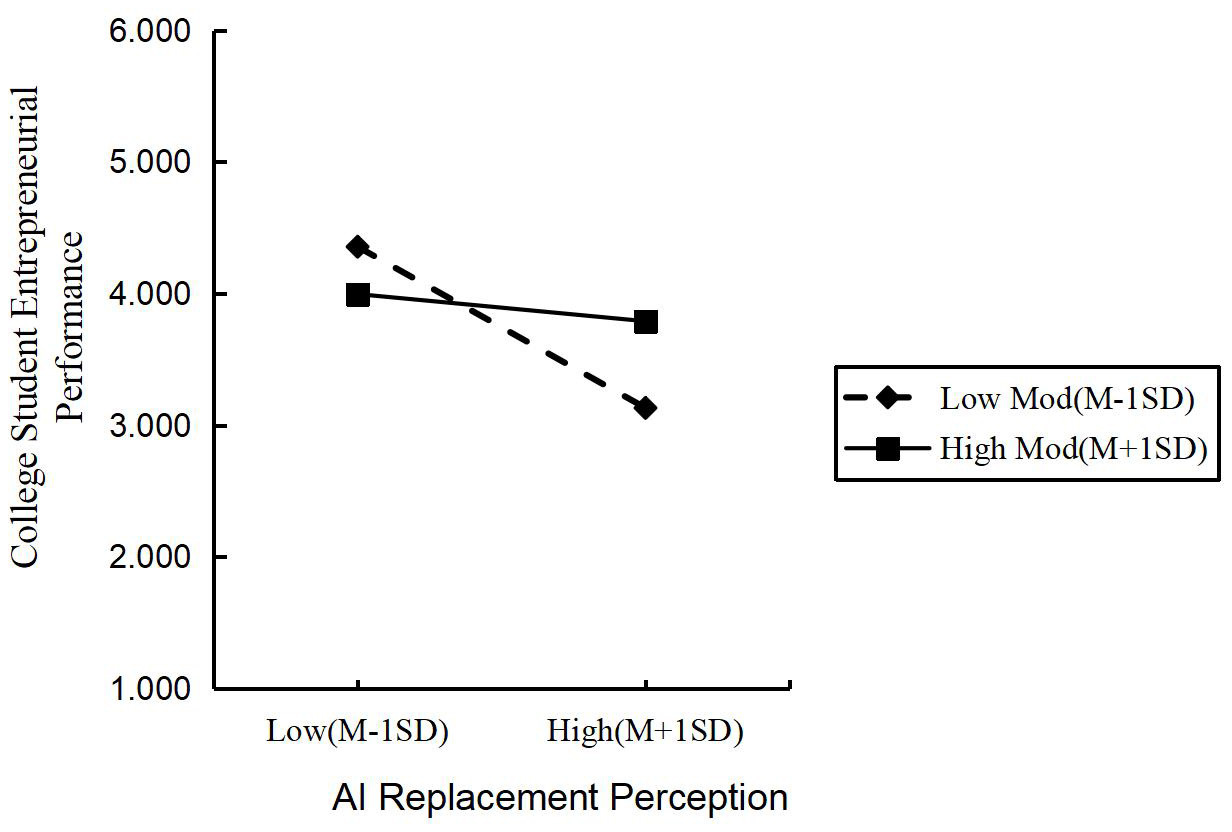
Moderating effect of VCP on the AIRP–CSEP relationship.

To further interpret the moderating effects, simple slope analyses were conducted and plotted in Figures 2, 3. As shown in Figure 2, the positive relationship between AI self-efficacy and college student entrepreneurial performance became stronger as value co-creation participation increased. As shown in Figure 3, the negative relationship between AI replacement perception and college student entrepreneurial performance became weaker as value co-creation participation increased.

### 4.4 Robustness tests

#### 4.4.1 Heteroskedasticity-robust standard errors

To examine whether the regression results were affected by potential heteroskedasticity, this study re-estimated the main models using heteroskedasticity-robust standard errors. As shown in Table 7, the results remained substantively consistent with the baseline findings. In Model (1), Gen AI had a significant positive effect on college student entrepreneurial performance (β = 0.399, *p* < 0.01). AI self-efficacy also had a significant positive effect on college student entrepreneurial performance (β = 0.296, *p* < 0.01), whereas AI replacement perception had a significant negative effect on college student entrepreneurial performance (β = −0.428, *p* < 0.01). These findings indicate that the main effect and the two psychological pathways remained stable after correcting for potential heteroskedasticity.

**Table 7:** Robustness tests.

Variables	(1)	(2)	(3)	(4)	(5)
	CSEP	Growth	Innovation	Growth	Innovation
GAI	0.399^***^	0.429^***^	0.369^***^	0.441^***^	0.379^***^
	0.045	0.049	0.046	0.045	0.042
AISE	0.296^***^	0.288^***^	0.304^***^		
	0.052	0.056	0.054		
AIRP	−0.428^***^	−0.436^***^	−0.420^***^		
	0.052	0.057	0.054		
c_AISE				0.300^***^	0.310^***^
				0.057	0.055
c_AIRP				−0.322^***^	−0.310^***^
				0.060	0.056
c_vcp				0.091^**^	0.102^**^
				0.045	0.042
AISE x VCP				0.110^***^	0.096^**^
				0.042	0.041
AIRP x VCP				0.268^***^	0.252^***^
				0.050	0.048
N	376	376	376	376	376
R2	0.531	0.495	0.495	0.551	0.548
Δ R^2^	0.509	0.471	0.471	0.526	0.522

c_AISE, c_AIRP, and c_VCP denote the mean-centered variables of AI self-efficacy, AI replacement perception, and value co-creation participation, respectively. AISE × VCP and AIRP × VCP denote the interaction terms constructed from the mean-centered variables. Growth denotes entrepreneurial growth performance, and Innovation denotes entrepreneurial innovation performance. Control variables include gender, age, competition track, competition level, and task responsibility. ^**^p < 0.05, ^***^p < 0.01.

#### 4.4.2 Task-role categorical controls

Considering that students undertake different responsibilities within CICSIC teams, task role may influence students' perceived entrepreneurial performance. For example, students responsible for business operations, market analysis, financial and legal affairs, technical development, or project presentation may differ in their level of involvement, perceived contribution, and visibility within the team. To reduce the possibility that the findings are driven by task-role differences, this study treated task responsibility as a categorical control variable in the robustness models. As shown in Table 7, after controlling for task-role heterogeneity, the main conclusions remained unchanged. Gen AI remained positively associated with college student entrepreneurial performance and its two subdimensions. AI self-efficacy remained positively associated with performance outcomes, while AI replacement perception remained negatively associated with performance outcomes. The interaction effects involving value co-creation participation also remained significant. These results suggest that the findings are not merely attributable to differences in students' task responsibilities within entrepreneurial teams.

#### 4.4.3 Alternative dependent variables

To further test whether the findings were driven by a single dimension of college student entrepreneurial performance, this study replaced the overall dependent variable with its two subdimensions: entrepreneurial growth performance and entrepreneurial innovation performance. Entrepreneurial growth performance, labeled as Growth in Table 7, refers to students' perceived improvement in entrepreneurial competence, entrepreneurial learning, and personal development through participation in CICSIC projects. Entrepreneurial innovation performance, labeled as Innovation in Table 7, refers to students' perceived innovation-related outcomes, such as improvements in creative thinking, project innovation quality, and innovative entrepreneurial achievements. Together, these two dimensions constitute the overall construct of college student entrepreneurial performance.

The results of Models (2) and (3) show that Gen AI had significant positive effects on both entrepreneurial growth performance (β = 0.429, *p* < 0.01) and entrepreneurial innovation performance (β = 0.369, *p* < 0.01). AI self-efficacy was also positively associated with both Growth (β = 0.288, *p* < 0.01) and Innovation (β = 0.304, *p* < 0.01), whereas AI replacement perception was negatively associated with both Growth (β = −0.436, *p* < 0.01) and Innovation (β = −0.420, *p* < 0.01). Models (4) and (5) further show that the moderating effects remained stable when the dependent variable was replaced. Specifically, the interaction between AI self-efficacy and value co-creation participation was significantly positive for both Growth (β = 0.110, *p* < 0.01) and Innovation (β = 0.096, *p* < 0.05). The interaction between AI replacement perception and value co-creation participation was also significantly positive for both Growth (β = 0.268, *p* < 0.01) and Innovation (β = 0.252, *p* < 0.01). These findings indicate that the conclusions are not driven by a single performance dimension but remain consistent across both entrepreneurial growth performance and entrepreneurial innovation performance.

## 5. Research conclusions and implications

### 5.1 Research conclusions

Drawing on the CICSIC context, this study examines how Gen AI is associated with college students' self-reported entrepreneurial growth and innovation performance in team-based competition projects. Based on a three-wave time-lagged survey of 376 valid respondents, the findings indicate that Gen AI has a significant positive effect on college student entrepreneurial performance. The results further reveal two appraisal-related perceptual pathways. First, Gen AI enhances AI self-efficacy, which in turn promotes entrepreneurial performance, suggesting an enabling pathway through which students' confidence in using Gen AI supports their perceived entrepreneurial learning and innovation outcomes. Second, Gen AI increases AI replacement perception, which subsequently weakens entrepreneurial performance, suggesting a constraining pathway in which perceived replacement threat undermines students' entrepreneurial outcomes. In addition, value co-creation participation serves as an important boundary condition by strengthening the positive relationship between AI self-efficacy and entrepreneurial performance while weakening the negative relationship between AI replacement perception and entrepreneurial performance.

### 5.2 Theoretical contributions and practical implications

#### 5.2.1 Theoretical contributions

First, this study extends research on Gen AI in higher education from general learning and academic support contexts to competition-based entrepreneurship education. By focusing on the CICSIC, this study explains how Gen AI may influence students' entrepreneurial growth and innovation performance in authentic entrepreneurial project tasks.

Second, in response to the call by Malik et al. for further research on the dual parallel mediation mechanisms underlying AI-related perceptions (Malik et al., 2022), this study applies cognitive appraisal theory of stress to explain the double-edged psychological mechanism through which Gen AI influences college student entrepreneurial performance. Specifically, AI self-efficacy is conceptualized as a challenge-oriented appraisal outcome, reflecting students' perceived capability and confidence in using Gen AI to cope with competition-related entrepreneurial tasks. AI replacement perception is conceptualized as a threat-oriented appraisal outcome, reflecting students' perceived risk that Gen AI may weaken their competence, task contribution, team role, or future entrepreneurial value.

Third, this study identifies value co-creation participation as a collaborative boundary condition. Value co-creation participation is treated as a contextual condition rather than an independent theoretical pillar. It helps explain why AI self-efficacy may be more strongly translated into entrepreneurial performance and why the negative effect of AI replacement perception may be weakened in collaborative human–AI and team-based environments.

#### 5.2.2 Practical implications

First, this study finds that the effect of Gen AI on college student entrepreneurial performance is not simply a matter of the more, the better (Klingbeil et al., 2024). Rather, Gen AI influences performance through the joint operation of AI self-efficacy and AI replacement perception. This finding suggests that team leaders and faculty advisors should move beyond generic technical training and adopt differentiated guidance strategies according to students' varying perceptions of AI. For students with high AI self-efficacy who actively embrace Gen AI, guidance should not be limited to operational training (Chen et al., 2019). Instead, these students should be encouraged to gain practical experience through competition participation and market-oriented project refinement. At critical stages such as business model design and core logic development, advisors may introduce regular “AI-free reflection sessions,” requiring team members to first develop and refine their ideas independently before comparing them with AI-generated suggestions (Yang and Jiang, 2025). Such practices can help ensure that the efficiency gains brought by Gen AI do not come at the expense of critical thinking, originality, and independent judgment.

Second, this study shows that Gen AI may reduce entrepreneurial performance through AI replacement perception. This finding indicates that competition organizers and university innovation and entrepreneurship associations should establish intervention mechanisms that combine pre-competition guidance with post-competition support in order to mitigate the negative effects of threat appraisal (Zong et al., 2025). For example, before the competition, previous award winners and expert panels could be invited to deliver AI-themed lectures. Such activities would not only create an active pre-competition atmosphere but also help participants better understand the ethical risks and appropriate boundaries of AI use. Moreover, the boundary-condition effect of value co-creation participation found in this study suggests that alleviating AI replacement perception depends primarily on interpersonal interaction rather than isolated human–machine interaction. Therefore, post-competition peer-support mechanisms are indispensable. Colleges may organize peer mentoring programs to help participating students address the innovation and entrepreneurship challenges encountered during the competition, foster a mentoring culture in which senior students support junior students, and facilitate timely reflection on the strengths and weaknesses of the competition process. For students who already exhibit signs of AI replacement perception, teams should provide targeted value clarification by emphasizing the irreplaceable human elements embedded in entrepreneurial projects. Through differentiated guidance based on students' varying levels of AI perception, team leaders and faculty advisors can transform Gen AI from a potential source of pressure into a genuine collaborative resource (Düz and Kavak, 2026).

Finally, the CICSIC is a team-based competition, and participating students should actively engage in value co-creation among humans, machines, and team members. For participants, the key is to recognize that the most effective role of Gen AI is not to function as an autonomous problem solver but to serve as a collaborative tool whose value depends on critical evaluation and responsible use. Individual participants should not only strengthen their AI application skills but also continuously improve their own capabilities, acquire disciplinary knowledge, enhance professional competence, and fulfill their specific responsibilities within the team. At the same time, they should develop stronger communication skills, seize key entrepreneurial opportunities, seek funding, source projects, and recruit high-caliber talent for the team.

#### 5.2.3 Future work

First, this study did not fully account for heterogeneity among participating college students in terms of their prior experience with Gen AI and entrepreneurial capabilities. Future research could examine the differentiated manifestations of Gen AI's double-edged sword effect along a “novice–intermediate-expert” continuum (Huang et al., 2025). In the context of the CICSIC, students with different levels of experience may exhibit distinct cognitive appraisal patterns and behavioral responses toward Gen AI. For novices, namely students who encounter Gen AI for the first time and lack prior experience in entrepreneurship competitions, the automation functions of Gen AI may rapidly lower entry barriers, enabling them to achieve visible outcomes in the short term and thereby develop a strong sense of AI self-efficacy. However, precisely because they lack the ability to critically evaluate AI-generated content, novices may also fall into overreliance without being fully aware of it. For intermediate students who already possess some competition experience and AI usage experience, the functional boundaries and limitations of Gen AI may be clearer. As a result, they may be better able to allocate cognitive resources between AI assistance and independent judgment. Nevertheless, this group may also face more subtle risks. As they transition from “knowing how to use AI” to “using AI effectively,” they may experience a stronger sense of replacement if they find that AI-generated outputs surpass their own original ideas during key creative stages. In such cases, their established sense of efficacy may be weakened, and AI replacement perception may increase rather than decrease. For expert students with both deep technical expertise and extensive entrepreneurial experience, Gen AI is more likely to be regarded as a convenient collaborative tool. Although these students may have internalized relative mature human AI collaboration strategies, they should also remain alert to the possible rigidification of innovation pathways caused by technological inertia.

Second, another limitation is that college student entrepreneurial performance was measured using self-reported data. Although self-reported performance is suitable for capturing individual students' perceived entrepreneurial growth and innovation outcomes, it may involve subjective bias. Future research could combine self-reported data with objective competition indicators, such as award level, advancement status, judges' scores, or project ranking, to provide a more comprehensive evaluation of entrepreneurial performance in competition-based educational contexts.

Finally, another limitation concerns the potential nested structure of the data. The CICSIC is a team-based competition, and some respondents may have come from the same competition teams due to the use of snowball sampling. Because the questionnaire was administered anonymously and did not collect identifiable team codes, this study was unable to calculate intraclass correlation coefficients or conduct multilevel modeling to formally assess team-level clustering effects. Although all focal constructs were measured at the individual perception level, future research should collect non-identifiable team identifiers and use multilevel modeling to distinguish individual-level effects from team-level influences. This would help clarify whether team-level value co-creation climate further shapes the relationship between Gen AI use and college student entrepreneurial performance (China International College Students' Innovation Competition, 2025).

## Data Availability

The original contributions presented in the study are included in the article/Supplementary material, further inquiries can be directed to the corresponding author.
