# Supplementary material for: Study on the double-edged sword effect of gen AI on college student entrepreneurial performance: evidence from the CICSIC
Source: Front Psychol. 2026 Aug 5;17:1909353. doi: 10.3389/fpsyg.2026.1909353 (PMC13487580; doi:10.3389/fpsyg.2026.1909353)
Supplement: Supplementary file 1 [file Data_Sheet_1.docx]

**Appendix A. Measurement Items**

All measurement items were assessed using a five-point Likert scale ranging from 1 = strongly disagree to 5 = strongly agree.

**A.1 Gen AI: Complementarity of Gen AI**

| **Code** | **Item** |
| --- | --- |
| GAI1 | Gen AI provides effective resources that supplement my learning, work, and competition-related entrepreneurial tasks. |
| GAI2 | Gen AI helps me find resources that complement my task responsibilities in the competition project. |
| GAI3 | The resources provided by Gen AI, together with my existing abilities and knowledge, support my completion of competition-related tasks. |

**A.2 Gen AI: Utilization of Gen AI**

| **Code** | **Item** |
| --- | --- |
| GAI4 | By using Gen AI, I can easily obtain the resources I need, such as information or suggestions for solving problems. |
| GAI5 | By using Gen AI, I can quickly find the resources I need, such as information or suggestions for problem-solving. |
| GAI6 | I think that the resources obtained through Gen AI can be effectively used in my learning and competition-related tasks. |

**A.3 Gen AI: Value-Creation Benefit of Gen AI**

| **Code** | **Item** |
| --- | --- |
| GAI7 | Gen AI saves me time and effort when completing learning and competition-related tasks. |
| GAI8 | Gen AI makes it easier for me to obtain the information or content I need for learning and project development. |
| GAI9 | Gen AI brings great convenience to my learning and competition-related entrepreneurial tasks. |

**A.4 Gen AI: Heterogeneity of Gen AI**

| **Code** | **Item** |
| --- | --- |
| GAI10 | Compared with other technologies, the resources provided by Gen AI are difficult to imitate. |
| GAI11 | Compared with other technologies, the resources provided by Gen AI are difficult to replace. |

**A.5 AI Self-Efficacy**

| **Code** | **Item** |
| --- | --- |
| AISE1 | I am confident in using Gen AI even if no one is assisting. |
| AISE2 | I have enough skills to use Gen AI. |
| AISE3 | I feel confident using Gen AI even by referring only to online guidance such as prompt manuals or online help. |
| AISE4 | I feel confident when using Gen AI. |

**A.6 AI Replacement Perception**

| **Code** | **Item** |
| --- | --- |
| AIRP1 | I think that the work or tasks I take on in study and competition may be replaced by AI technology. |
| AIRP2 | I worry that the specific things currently done in learning and competition will be replaced by AI technology. |
| AIRP3 | As AI technology replaces the relevant work of participants, I am personally worried about my future development in the participating team. |
| AIRP4 | As AI technology replaces the relevant work of participants, I personally worry about my future participation in the field of competition. |

**A.7 Value Co-Creation Participation: Team Complementary Value Co-Creation**

| **Code** | **Item** |
| --- | --- |
| VCP1 | I am able to use Gen AI to communicate with my team. |
| VCP2 | The team can help us build an intelligent innovation and entrepreneurship ecosystem. |
| VCP3 | I can use Gen AI to jointly develop intelligent entrepreneurial projects with the team. |
| VCP4 | The intelligent service I provide requires the participation of team members. |
| VCP5 | The team can provide technical support for us to achieve intelligent operations. |

**A.8 Value Co-Creation Participation: Human-Machine Collaborative Value Co-Creation**

| **Code** | **Item** |
| --- | --- |
| VCP6 | Gen AI can help us achieve flexible innovation. |
| VCP7 | Gen AI can present us with creative ideas. |
| VCP8 | We can use the intelligent services provided by the competition platform to improve the competition experience. |

**A.9 College Student Entrepreneurial Performance**

| **Code** | **Item** |
| --- | --- |
| CSEP1 | Entrepreneurship has strong profitability and can generate considerable investment returns. |
| CSEP2 | In the process of innovation and entrepreneurship, I have a high utilization rate of funds, and my entrepreneurial operation is in good condition. |
| CSEP3 | The creative and technological achievements I have achieved in the process of innovation and entrepreneurship have a high success rate of transforming into products, and it is expected that they can be utilized by schools and the market. |
| CSEP4 | In the process of innovation and entrepreneurship, I can cooperate with team members to promote the development of personal relationship networks and expand the scale of entrepreneurship. |
| CSEP5 | I will be satisfied that I have achieved the goals I set at the beginning of my business. |
| CSEP6 | In the process of innovation and entrepreneurship, I used the content I learned from entrepreneurship education, strengthened my innovation awareness and entrepreneurial ability, and improved my overall quality. |

**A.10 Control Variables**

| **Variable** | **Categories** |
| --- | --- |
| Gender | Male; Female |
| Grade level | Freshman; Sophomore; Junior; Senior; Fifth year of university; Postgraduate |
| Competition track | Higher Education Main Track; Youth Red Dream Journey Track; Industry-Specific Track; Vocational Education Track |
| Competition level | University-level preliminary competition; Provincial semi-finals; Finals |
| Task responsibility within the team | Project manager; Technology research/core innovation; Business operations/market analysis/finance/legal affairs; Copywriting/material organization and reporting |
